# How to teach person-centered medicine during the coronavirus disease 2019 pandemic?

**DOI:** 10.3325/cmj.2022.63.98

**Published:** 2022-02

**Authors:** Marijana Braš, Veljko Đordević, Neda Pjevač, Snježana Kaštelan, Marijan Klarica, Slavko Orešković

**Affiliations:** 1 Center for Palliative Medicine, Medical Ethics and Communication Skills, University of Zagreb School of Medicine, Zagreb, Croatia *marijana.bras1@gmail.com*; 2 Department of Medical Statistics, Epidemiology and Medical Informatics, University of Zagreb, School of Medicine, Andrija Štampar School of Public Health, Zagreb, Croatia; 3 Department of Ophthalmology, University Hospital Dubrava, University of Zagreb School of Medicine, Zagreb, Croatia; 4 Department of Pharmacology, Zagreb University School of Medicine, Zagreb, Croatia; 5 Department of Obstetrics and Gynecology, University Hospital Center Zagreb, University of Zagreb School of Medicine, Zagreb, Croatia

Personalized medicine refers to sophisticated diagnostic and therapeutic interventions tailored to the patient's individual needs. The development of personalized medicine in the last two decades created a global movement of person-centered medicine and health care. Prominent medical organizations and institutions that advocate a personalized approach to medicine established an international network called the International College of Person-Centered Medicine (1,2).

Person-centered medicine emphasizes that the patient is a person with a biological, psychological, social, and spiritual dimension in health and disease. We need to understand all of these dimensions, as well as the risk factors involved, to be able to properly treat the patient as an individual and not merely as a disease. This holistic care paradigm highlights the importance of life perspective, personal values, and choices, as well as the context in which the patient lives.

The School of Medicine at the University of Zagreb has a tradition of adopting a holistic approach in medicine. However, introduction of teaching of person-centered medicine in the curriculum necessitated the reorganization of training and education of health care professionals (3,4). A significant step forward was the establishment of the Centre for Palliative Medicine, Medical Ethics and Communication Skills as a separate organizational department responsible for research, education, and training in these three domains (5). Many generations of students have been educated according to the person-centered model, particularly through courses related to communication skills, palliative care, and ethics in medicine. Teaching communication skills has become a requirement of post-graduate training for all specializations in Croatia. Given that these skills are acquired through practice and experiential learning, an interactive and personalized approach to teaching was adopted (6). Aiming to improve the quality of the existing medical practice, over the past ten years the School of Medicine has gradually introduced the Calgary Cambridge Medical Interview Model intended for both medical students and health care professionals (7). The COVID-19 pandemic brought challenges related to teaching of person-centered medicine. The aim of this article is to describe our experience of teaching communication skills and palliative medicine during the pandemic.

## Teaching communication skills during the COVID-19 pandemic

During the COVID-19 pandemic and after the earthquakes that struck our country in 2020 and 2021, we swiftly moved to online teaching, while nurturing and further developing person-centered medicine. The transfer was facilitated by support at the institutional level, flexible staff, and solid leadership. The longitudinal course Fundamentals of Medical Skills, continuing through all six years of the undergraduate program, offered a variety of exercises in communication skills via internet platforms. We continued to apply the “patient as a teacher” model. This model enables experiential learning through work with simulated or real patients with the aid of video materials. Some of these real patients experienced a severe COVID-19 infection and were even mechanically ventilated. During the online teaching, one student stated “After six years of studying, I have met the art of medicine and communicated with various patients who have taught me not only about their diseases and life experiences, but also showed me the beauty of life. I realize that people with diseases value life more than healthy people. I think that person-centered approach is a key to better medical care of the whole community.”

Attention has also been given to the mental health status of health care professionals due to the severe stress they have experienced during the pandemic. We continually emphasized that the relationships in medicine should be those of mutual trust and respect, and that patients should be treated as individuals rather than as diagnoses or symptoms (8). On the internet platform, we have taught out students how to work and communicate in a medical team. The emphasis was placed on communication with patients as a two-way road, in which both sides go their own way but are constantly communicating. Otherwise, no understanding can be reached. We have taught our students that it is better to give than to receive, whereby helping others may be the key to living a happier, healthier, more meaningful, and fulfilling life.

## Teaching person-centered palliative medicine during the COVID-19 pandemic

Palliative medicine has recently become a compulsory course for sixth-year students of integrated medical studies at the University of Zagreb School of Medicine. Additionally, a Department of Palliative Medicine has been formed. Lecturers at the department include specialists in oncology, psychiatry, neurology, surgery, family medicine, and anesthesiology, as well as graduate nurses, psychologists, social workers, and pharmacists, which reflects the interdisciplinary character of palliative medicine. Person-centered palliative medicine is promoted in each part of the course, and all our lecturers are trained in this discipline.

A real challenge was how to organize online teaching for medical students relating to patients who suffer from incurable diseases and their family members. We conducted classes (lectures, seminars, and exercises) via platforms such as Zoom. An excellent innovation were our weekly “joint meetings” on Zoom in which all course lecturers participated together with students. The moderator usually asked other lecturers how it was emotionally for them to work with patients in palliative care, why they had chosen their profession, and how they prevented burnout. These virtual meetings lasted three hours and were positively rated by the students. Students were impressed to be approached by lecturers “as people with their own life stories” and to experience communication in an interdisciplinary team. Since students could not visit palliative care departments or do home visits with mobile palliative care teams, we organized online exercises with real patients suffering from incurable diseases or their family members, after they signed informed consent. A woman who lost a husband due to pancreatic cancer told the students “The palliative team, through helping my husband, has shown how important communication skills and empathy are. Why am I sharing my experiences with you? I believe that it's very important for medical students to learn the basics through the courses of communication and palliative medicine so that eventually, as doctors, they can understand the palliative care system and responsibly and ethically make assessments and decisions during the process of dying.”

We also produced several films depicting patients' life stories, which were recorded in their homes. Students were confronted with people who had incurable diseases, talked to family members of recently deceased patients, and discussed biological, psychological, social, and spiritual factors. We wanted the students to understand how they often can only treat and care for the patients, but not cure them. As the presented real-life stories provoked strong emotional reactions from the students, teachers taught them how to recognize their reactions and cope with them. A lot of attention was paid to empathy and emotions.

The interaction during online teaching resulted in a book of essays written by teachers and students. The book Stand by Me, published in July 2021, is a real treasure trove of experiences and skills on how to best approach patients and family members in palliative medicine. Many authors also described the importance of helping and caring for ourselves as professionals. The students rated this course as one of the best of the year, giving us an incentive to further improve our teaching methods.

One of our teachers, a master of nursing, wrote: “A few months after the beginning of the pandemic, it was necessary to adjust teaching in new ways, namely bring in e-learning. We were enthusiastic and proud to participate in introducing a new course during the pandemic and after the earthquake that struck Zagreb and our School. However, I had many questions considering how to adjust to distance learning, interest the students into a currently unpopular field of medicine, and transfer my knowledge and experience from my position of the only master of nursing in this course: What is expected from me? What do I expect from myself? What do I expect from students? How am I going to adapt to the new, changeable conditions? Am I able to recognize the needs and interests of students? How to maintain and increase the interests of students, as well as the quality of teaching? Do I have enough knowledge and digital competencies? Will I be able to manage the new teaching platforms in a short period of time? Which ways of teaching are effective? How to evaluate acquired knowledge? Eventually, the education of students became the real team exchange of knowledge and experiences. Throughout conversations, examples of good practice, and discussions, I believe that we have brought palliative care closer to students and in doing so, got to know each other better.” (9).

We believe that our ten-year effort to educate thousands of students and professionals in palliative medicine has resulted in a great achievement. The greatest benefit was that our former students found it easier to work in COVID departments, repeatedly pointing out how the attained knowledge helped them to cope better. In fact, it turned out that the introduction of the Palliative Medicine course and a longitudinal education on communication skills is the best way to create a doctor who will deal with this difficult situation.

## Conclusion

The twentieth-century medicine branched into many specializations and subspecializations within each profession, while the twenty-first century medicine will move toward integration. As a result, we need to open up to a new ecology of the mind (10). This goal can only be achieved by health professionals who were educated according to the model of person-centered medicine and health. This was particularly evident at the beginning of the COVID-19 pandemic, which required a rapid change in teaching methods, the introduction of distance learning, and teacher flexibility.

Today’s world is at a civilizational turning point, but this time of crisis is a historic opportunity to create new models of education for future professionals, who will need to cope with numerous challenges in their work. These models should already be incorporated into medical education. Knowledge is not knowledge to have but knowledge to give, and this is our path. The COVID-19 pandemic accelerated the process of transformation. We need an international network of teachers who will create new medical curricula by taking into account person-centered medicine. We will use our experience from the last two years to improve teaching. We will also continue to pay great attention not only to the education of future professionals, but also to the education of the current teachers, with an emphasis on a teaching methodology that can follow the trends.
